# Should the Prevalence of Incidental Thyroid Cancer Determine the Extent of Surgery in Multinodular Goiter?

**DOI:** 10.1371/journal.pone.0168654

**Published:** 2016-12-22

**Authors:** Krzysztof Kaliszewski, Marta Strutyńska-Karpińska, Agnieszka Zubkiewicz-Kucharska, Beata Wojtczak, Paweł Domosławski, Waldemar Balcerzak, Tadeusz Łukieńczuk, Zdzisław Forkasiewicz

**Affiliations:** 1 1st Department and Clinic of General, Gastroenterological and Endocrine Surgery, Wroclaw Medical University, Wroclaw, Poland; 2 Department of Endocrinology and Diabetology for Children and Adolescents, Wroclaw Medical University, Wroclaw, Poland; Fu Jen Catholic University, TAIWAN

## Abstract

**Background:**

The most appropriate surgical procedure for multinodular goiter (MNG) remains under debate. Incidental thyroid carcinoma (ITC) is often identified on histopathological examination after thyroidectomy performed for presumed benign MNG.

**Aim of the study:**

The aim of the study was to determine the value of radical surgery for MNG patients considering the prevalence of ITC diagnosed postoperatively.

**Materials and Methods:**

We conducted retrospective analysis of the medical records of 2,306 patients surgically treated for MNG between 2008 and 2013 at one center. None of the patients presented with any suspicion of malignancy, history of familial thyroid cancer, multiple endocrine neoplasia syndrome or previous head or neck radiation exposure.

**Results:**

Among the 2,306 MNG patients, ITC was detected in 49 (2.12%) (44 women and 5 men, with average ages of 52.2 (21–79) and 55.6 (52–62), respectively). Papillary thyroid carcinoma was significantly more frequently observed than other types of ITC (p<0.00001). Among the MNG patients, 866 (37.5%) underwent total/near total surgery, 464 (20.1%) received subtotal thyroidectomy, and 701 (30.3%) received the Dunhill operation. The remaining 275 (11.9%) patients underwent a less radical procedure and were classified as "others." Among the 49 (100%) patients with ITC, 28 (57.1%) underwent radical surgery. Another 21 (42.9%) patients required completion surgery due to an insufficient primary surgical procedure. A total of 21 (2.42%) patients in the total/near total surgery group were diagnosed with ITC, as well as 16 (2.48%) in the subtotal thyroidectomy group and 12 (1.71%) in the Dunhill operation group; 21 (100%), 4 (25%) and 3 (25%) of these patients, respectively, underwent radical surgery; thus, 0 (0%), 12 (75%) and 9 (75%) required completion surgery. The prevalence rates of ITC were comparable between the radical and subtotal surgery groups (2.42% and 3.44%, respectively, p = 0.4046), and the prevalence was higher in the radical surgery group than in the Dunhill operation group (2.42% and 1.71%, respectively, p = 0.0873). A significant difference was observed between the group of patients who underwent total/near total surgery, among whom all of the patients with ITC (100%) received primary radical surgery, and the groups of patients who received the subtotal and Dunhill operations, among whom only 25% of the patients with ITC in each group received primary radical surgery (p<0.0001).

**Conclusions:**

More radical procedures for MNG result in a lower risk of reoperation for ITC. The prevalence of ITC on postoperative histopathological examination should determine the extent of surgery in MNG patients. In the future, total/near total thyroidectomy should be considered for MNG patients due to the increased prevalence of ITC to avoid the necessity for reoperation.

## Introduction

The prevalence of incidental thyroid cancer (ITC) in multinodular goiter (MNG) has been previously estimated to be 5–10% [1–3]; however, recent studies have reported higher ITC prevalence rates, ranging from 8.6 to 22% [4–7]. Further, the detection rate of ITC on autopsy examination has been reported to be steadily rising, with an estimated increase from 6% in 2003 to 20% in 2012 [8]. This perceived increase might have been a consequence of the high prevalence of thyroid nodules detected in the autopsy series of 50% [5–7]. Bae et al. [9] have found that the prevalence of malignancy in patients with incidentally identified thyroid lesions on F-fluoro-deoxyglucose positron emission tomography examination is higher than 23%. In addition, the use of high-resolution ultrasonography has been reported to detect asymptomatic thyroid nodules in 13% of studied patients, with a malignancy rate among these lesions of 29% [10].

A common clinical scenario is the incidental finding of thyroid carcinoma (TC) during histopathological examination after strumectomy performed for presumed benign MNG. Thus, a clinical dilemma exists regarding the decision of the extent of MNG surgery that should be performed considering ITC occurrence [11], and it requires special consideration and multidisciplinary management.

The optimal surgical procedure for MNG patents remains a subject of debate, due to not only the increasing prevalence of ITC among these patients but also the incidence of TC in recurrent goiter [7]. Total thyroidectomy is the clear procedure of choice for the treatment of most TCs; however, there is currently no surgical recommendation for MNG management that takes into account the prevalence of ITC. Some authors have indicated that total thyroidectomy should be performed for MNG, especially that in endemic iodine-deficient regions [12–15]. Disadvantages of subtotal thyroidectomy for MNG include the increased prevalence of ITC in this thyroid pathology and the high recurrence rate after this procedure observed during long-term follow-up [13–19]. Some authors have reported that the recurrence rate after subtotal thyroidectomy performed for MNG is approximately 50%; however, this rate depends on the duration of observation [20]. In this particular study, the authors observed the patients for approximately 180 months. Many researchers have emphasized the disadvantage of leaving thyroid tissue in the operative bed after subtotal thyroidectomy [21–24]. They have suggested that in the future, some of these patients may require reoperation due to ITC in recurrent goiter, which may be associated with a worse prognosis and a higher rate of complications compared with patients who have received primary surgery [21–23]. John et al. [24] examined 1300 thyroidectomy specimens for the presence of incidental papillary thyroid microcarcinoma. These authors noted that the most common indication for surgery among this group of patients was MNG with consideration of the ITC prevalence. They found 94 cases of papillary thyroid microcarcinoma, although malignancy was suspected in only 31.4% of the cases.

The presence of multiple nodules in the thyroid gland decreases the diagnostic value of some preoperative examinations, including fine-needle aspiration biopsy (FNAB), resulting in detection of TC on postoperative histological examination in many patients [25]. In such cases, reoperation after non-radical procedures (e.g., subtotal or Dunhill operation) is necessary. Moreover, some authors have indicated that papillary thyroid microcarcinoma is a rather common incidental finding after surgery performed for MNG [26]. The presence of one solitary thyroid nodule in MNG is generally considered to be associated with a higher risk of malignancy than the presence of multiple nodules. Therefore, only such lesions should be subjected to FNAB, which would increase the diagnostic value of this procedure. The strategy is not as clear for patients with many coexisting nodules [25]. A lesion that has grown progressively, has become dominant or has changed in consistency should be biopsied. Tollin has suggested that such nodules carry the same risk of malignancy as a solitary nodule [1]. This finding may increase the accuracy of MNG diagnosis and thereby inform decision making regarding the extent of surgery. In cases of MNG without a dominant nodule, the most important consideration for optimizing therapeutic decision making is whether the process is malignant or benign. Obviously, total thyroidectomy is the ideal surgical procedure for the treatment of TC diagnosed before surgery. However, if thyroid malignancy is detected on postoperative histopathology after a less radical procedure has been performed, then a subsequent completion procedure is often mandatory [27]. The same authors have concluded that the best procedure for treatment of ITC is total or near total thyroidectomy, especially if the malignant foci are bilateral. A completion procedure is indicated if the initial surgery was partial or subtotal.

The aim of this study was to determine the value of total/near total thyroidectomy in MNG patients considering the prevalence of ITC diagnosed postoperatively and the need for completion surgery.

## Materials and Methods

The agreement for our study was prepared and approved by the Bioethics Committee of Wroclaw Medical University (signature number: KB-296/2015). We obtained oral consent from our participants. The data were analyzed anonymously on the basis of medical records. The authors did not have access to patient identifying information or direct access to the study participants.

The authors retrospectively analyzed the medical records and histopathological reports of 2,306 patients who were admitted to and surgically treated for presumed benign MNG at the 1^st^ Department and Clinic of General, Gastroenterological and Endocrine Surgery of Wroclaw Medical University (Poland) from 1^st^ January, 2008, to 31^st^ December, 2013. Analysis was conducted to determine the number of patients with ITC diagnosed on postoperative histopathological examination and to estimate the rates of completion surgery for ITC diagnosed after surgery for MNG. We compared the number of patients with ITC in each group according to the type of primary surgery performed for MNG. In accordance with the American Thyroid Association Guidelines from 2015, we accepted total and near total thyroidectomy as sufficient radical procedures for unifocal and unilateral ITC treatment.

The exclusion criteria were as follows: suspicion of malignancy before or during surgery or any preoperative diagnosis that determined the extent of the surgical procedure, even to a minimal degree (e.g., follicular neoplasm or atypia of undetermined significance/follicular lesion of undetermined significance (AUS/FLUS) on cytology). All of the included patients underwent clinical examination and routine thyroid and neck lymph node ultrasonography. Thyrotropin-stimulating hormone (TSH), thyroxine (fT4), triiodothyronine (fT3), antithyroid peroxidase/antithyroglobulin antibodies (anti-TPO/anti-Tg), thyroglobulin (Tg) and calcitonin levels were routinely measured. All 2,306 (100%) patients who initially qualified for our study underwent ultrasonography-guided FNAB (UG-FNAB). Scintigraphy and computed tomography (CT) were performed to determine the retrosternal extension of the goiter as necessary. All MNG patients who qualified for surgery and were enrolled in this study had a thyroid hormone level that was within the normal range, no signs or symptoms of thyroid malignancy, and no suspicion of malignancy. All qualified patients underwent one of the following three clearly defined surgical procedures: total/near total, subtotal or Dunhill operation. After surgery, routine histopathological examination of 1 mm sections of specimens obtained from all patients was performed using a serial sectioning technique. Among all studied individuals, those with a histopathological diagnosis of TC determined during postoperative examination were selected for further analysis. We assessed the histopathological types of malignancy, types of surgeries performed for MNG, numbers of ITC diagnoses made in each group and numbers of patients who required completion surgery after initial non-radical treatment.

### Statistical analysis

Statistical analysis was conducted using Statistica software (StatSoft, Inc. (2014) STATISTICA (data analysis software system), version 12, www.statsoft.com), licensed by the Wroclaw Medical University. The data were reported as the mean (x), median and standard deviation (SD), or range. The Shapiro-Wilk test was used to confirm the similarity of the age distribution of the analyzed samples with the normal distribution. As the distribution of the analyzed samples did not significantly differ from the normal distribution, with statistically identical variances, the t-test was used to assess eventual differences. Intergroup frequency was assessed using the chi-square test. Yate's correction was applied when the expected frequency was less than 5 or the total count was less than 50. Any difference with a p value of <0.05 was considered statistically significant, while p values ranging from 0.05 to <0.10 were considered to indicate borderline statistical significance.

## Results

**Fig 1:**
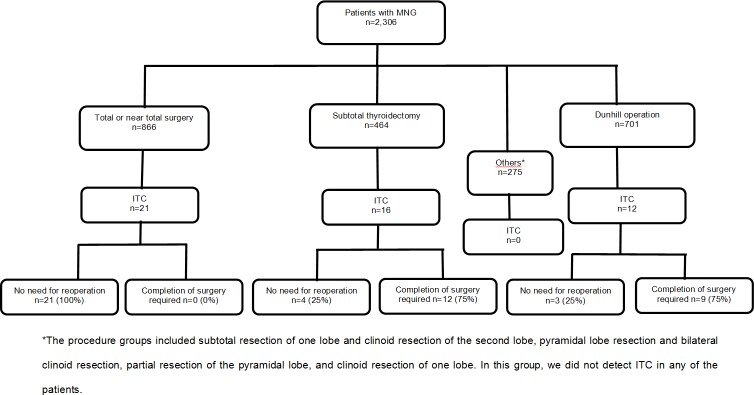
The prevalence of ITC according to each type of surgery for MNG.

**Fig 2:**
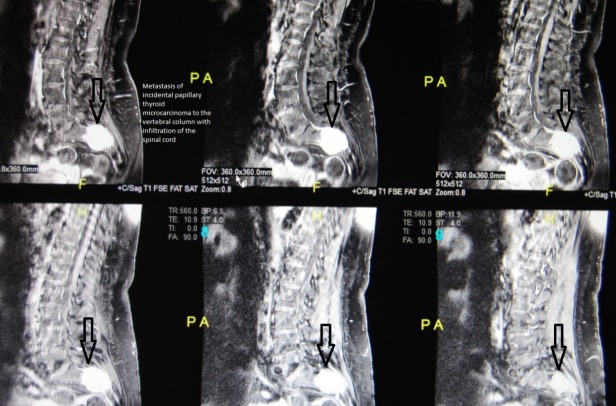
MRI sagittal scan showing area of high signal (arrows) corresponding to spinal metastasis of ITC in a 61-year-old female.

**Fig 3:**
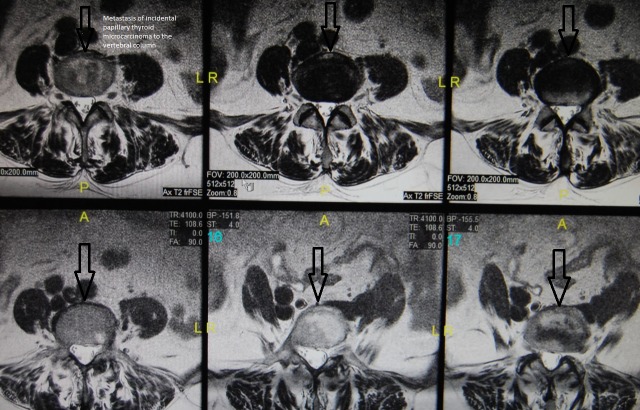
MRI transverse scan of the vertebral column showing a low signal area (arrows) of ITC metastasis in a 61-year-old female.

The prevalence of ITC was 2.12% among all patients who underwent surgery due to presumed benign MNG (Fig 1). A series of 49 patients (44 females and 5 males) with ITC is presented here. The mean patient age at the time of surgery was 52.6 years (52.2 (21–79) for females and 55.6 (52–62) for males, p = 0.6362). No cervical or mediastinal lymphadenopathy was detected in any of the patients before or during surgery. A total of 46 (93.8%) patients had papillary TC; among them, 43 (87.7%) had the classical variant, 3 (6.1%) had the follicular variant, 2 (4.0%) had the oxyphilic type of follicular TC and 1 (2.0%) had undifferentiated carcinoma. Three (6.1%) patients with incidental papillary TC were diagnosed with multifocal cancer (Table 1). Papillary TC was significantly more frequently detected than the other types of ITC (p<0.00001). Furthermore, among the patients with this type of ITC, the classical variant was the most commonly observed (p<0.00001). In one 61-year-old female patient who had undergone surgery in 2011, a classical variant of ITC was identified after diagnosis of ITC metastases to the vertebral column and spinal cord (Figs 2 and 3).

**Table 1:** Histopathological types of incidental thyroid cancer.

Histopathological type	Year						Total
	2008	2009	2010	2011	2012	2013	
Papillary thyroid carcinoma	7 (14.2%)	4 (8.1%)	5 (10.2%)	5 (10.2%)	13 (26.5%)	12 (24.4%)	46 (93.8%)
Classical variant	7 (14.2%)	4 (8.1%)	3 (6.1%)	5 (10.2%)	12 (24.4%)	12 (24.4%)	43 (87.7%)
Follicular variant	0 (0.0%)	0 (0.0%)	2 (4.0%)	0 (0.0%)	1 (2.0%)	0 (0.0%)	3 (6.1%)
*Multifocal	0 (0.0%)	1 (2.0%)	0 (0.0%)	1 (2.0%)	1 (2.0%)	0 (0.0%)	3 (6.1%)
*Microcarcinoma	5 (10.2%)	2 (4.0%)	4 (8.1%)	2 (4.0%)	8 (16.3%)	7 (14.2%)	28 (57.1%)
Follicular thyroid carcinoma	0 (0.0%)	0 (0.0%)	1 (2.0%)	0 (0.0%)	0 (0.0%)	1 (2.0%)	2 (4.0%)
Non-differentiated thyroid carcinoma	0 (0.0%)	0 (0.0%)	1 (2.0%)	0 (0.0%)	0 (0.0%)	0 (0.0%)	1 (2.0%)
Total	7 (14.2%)	4 (8.1%)	7 (14.2%)	5 (10.2%)	13 (26.5%)	13 (26.5%)	49 (100%)

*within classical/follicular variant papillary thyroid carcinoma

Total and near total thyroidectomy were the most common surgical procedures performed (Table 2). Among all of the MNG patients, 866 (37.5%) underwent total or near total surgery, 464 (20.1%) received subtotal thyroidectomy, and 701 (30.3%) underwent the Dunhill operation. Another 275 (11.9%) patients underwent less radical procedures; these patients were classified as "others" and included those who received subtotal resection of one lobe and clinoid resection of the second lobe, pyramidal lobe resection and bilateral clinoid resection, partial resection of the pyramidal lobe, and clinoid resection of one lobe. No ITC was identified in this group.

**Table 2:** Types of surgery among patients with multinodular goiter.

Year	Patients with MNG		Type of surgery			
	Women	Men	Total/Near total	Subtotal	Dunhill operation	Others*
2008	333 (96.2%)	13 (3.7%)	77 (22.2%)	156 (45.0%)	86 (24.8%)	27 (7.8%)
2009	288 (93.5%)	20 (6.4%)	78 (25.3%)	108 (35.0%)	113 (36.6%)	9 (2.9%)
2010	324 (91.0%)	32 (8.9%)	99 (27.8%)	102 (28.6%)	138 (38.7%)	17 (4.7%)
2011	341 (87.8%)	47 (12.1%)	180 (46.3%)	48 (12.3%)	142 (36.5%)	18 (4.6%)
2012	390 (89.4%)	46 (10.5%)	202 (46.3%)	33 (7.5%)	121 (27.7%)	80 (18.3%)
2013	429 (90.8%)	43 (9.1%)	230 (48.7%)	17 (3.6%)	101 (21.3%)	124 (26.2%)
Total	2105 (91.2%)	201 (8.7%)	866 (37.5%)	464 (20.1%)	701 (30.3%)	275 (11.9%)
	P < 0.00001		Total/Near total surgery was the most frequent (p<0.00001)			

*Surgeries less radical than the Dunhill and subtotal operations: subtotal resection of one lobe and clinoid resection of the second lobe, pyramidal lobe resection and bilateral clinoid resection, partial resection of the pyramidal lobe, and clinoid resection of one lobe.

The demographic data and staging of ITC according to the AJCC 2010 classification among the MNG patients are presented in Table 3.

**Table 3:** Demographic data and staging of incidental thyroid carcinoma according to the AJCC 2010 classification for patients with multinodular goiter.

	Radical surgery	Non-radical surgery	p
Number of patients, n (%)	28 (57.1%)	21 (42.9%)	0.2255
Mean age, years +/- (range)	53.3 +/-16.3 (21–79)	51.6 +/-13.4 (25–74)	0.6963
Age <45 years old, n (%)	8 (28.6)	7 (33.3)	0.9643
Age ≥45 years old, n (%)	20 (71.4)	14 (66.7)	
Gender, F:M	25:03:00	19:02	0.7334
Histopathological type, n (%)			
Papillary thyroid carcinoma	27 (96.4)	19 (90.5)	
Classical variant	26 (92.8)	17 (81.0)	*
Follicular variant	1 (3.6)	2 (9.5)	**
Follicular thyroid carcinoma	1 (3.6)	1 (4.7)	
Non-differentiated thyroid carcinoma	0 (0.0)	1 (4.7)	
TNM classification 2010, n (%)			
pT1a	18 (64.3)	11 (52.4)	p = 0.99743
pT1b	8 (28.6)	9 (42.8)	
pT2	2 (7.1)	1 (4.7)	
pT3	0 (0.0)	0 (0.0)	
pT4a	0 (0.0)	0 (0.0)	
pT4b	0 (0.0)	0 (0.0)	
pT(m)	2 (7.1)	1 (4.7)	
pNx	28 (100)	21 (100)	
pMx	28 (100)	21 (100)	
pTNM staging according to AJCC 2010, n (%)			
I	26	20	p = 0.7964
II	2	1	
III	0	0	
IV	0	0	

* Irrespective of the type of surgical procedure (radical vs. non-radical), the prevalence rates of papillary thyroid carcinoma and non-differentiated thyroid carcinoma were comparable: P = 0.8790

** Irrespective of the type of surgical procedure (radical vs. not-radical), the prevalence rates of the histopathological variants of papillary thyroid carcinoma were comparable: p = 0.97186

Radical surgery was performed to treat 28 (57.1%) of the patients with ITC, and the remaining 21 (42.9%) patients required completion surgery due to an insufficiently radical primary surgical procedure (p = 0.2255).

A total of 21 (2.42%) of the patients who underwent radical surgery were found to have ITC, in addition to 16 (2.48%) of those who received subtotal thyroidectomy and 12 (1.71%) of those who underwent the Dunhill operation (Table 4). Among these patients, 21 (100%), 4 (25%) and 3 (25%) underwent radical surgery; thus, 0 (0%), 12 (75%) and 9 (75%) required completion surgery, respectively. The prevalence of ITC was comparable between the groups of patients who underwent radical (total/near total) and subtotal surgery (2.42% and 3.44%, respectively, p = 0.4046). This prevalence was markedly higher in the group of patients who underwent radical (total/near total) surgery than in the group of those who received the Dunhill operation (2.42% and 1.71%, respectively, p = 0.0873). A significant difference was detected between the group of patients who underwent total or near total surgery, among whom all of the patients with ITC (100%) received primary radical surgery, and the other two groups of patients who underwent subtotal surgery and the Dunhill operation, respectively, among whom only 25% of the patients with ITC in each group received primary radical surgery (Table 5, p<0.0001). Reoperation was performed after histopathological examination for 21 (42.8%) of the patients who underwent non-radical thyroidectomy (Table 4).

**Table 4:** Incidental thyroid carcinoma in multinodular goiter.

Year	Incidental thyroid cancer		Type of surgery					
	Women	Men	Total or near total (I)	Subtotal (II)	Dunhill operation (III)	Radical	Non-radical	Completion of surgery
2008	7 (0.33%)	0 (0.00%)	0 (0.0%)	5 (71.4%)	2 (28.5%)	2 (28.5%)	5 (71.4%)	5 (71.4%)
2009	2 (0.10%)	2 (1.00%)	2 (50.0%)	1 (25.0%)	1 (25.0%)	2 (50.0%)	2 (50.0%)	2 (50.0%)
2010	6 (0.29%)	1 (0.50%)	0 (0.0%)	5 (71.4%)	2 (28.5%)	1 (14.2%)	6 (85.7%)	6 (85.7%)
2011	5 (0.24%)	0 (0.00%)	2 (40.0%)	3 (60.0%)	0 (0.0%)	2 (40.0%)	3 (60.0%)	3 (60.0%)
2012	11 (0.52%)	2 (1.00%)	7 (53.8%)	2 (15.3%)	4 (30.7%)	9 (69.2%)	4 (30.7%)	4 (30.7%)
2013	13 (0.62%)	0 (0.00%)	10 (76.9%)	0 (0.0%)	3 (23.0%)	12 (92.3%)	1 (7.6%)	2 (15.3%)
Total	44 (2.09%)	5 (2.49%)	21 (42.8%)	16 (32.6%)	12 (24.4%)	28 (57.1%)	21 (42.8%)	21 (42.8%)
			p_IvsII_ = 0.4046			P = 0.2255		
			p_IvsIII_ = 0.0873					
			p_IIvsIII_ = 0.5023					

**Table 5:** Type of procedure (radical/non-radical) according to necessity for completion of primary surgery (total or near total, subtotal, and Dunhill operation).

	Total or near total (I)	Subtotal (II)	Dunhill operation (III)
Radical	21(100.0%)	4 (25.0%)	3 (25.0%)
Non-radical	0 (0.0%)	12 (75.0%)	9 (75.0%)
Total	21 (42.8%)	16 (32.6%)	12 (24.4%)
	p_IvsII_ < 0.0001		
	p_IvsIII_ < 0.0001		
	p_IIvsIII_ = 0.6592		

The number of radical operations increased considerably during the analyzed time frame, from 22% in 2008 to 49% in 2013 (p = 0.0001). During those years, the prevalence of ITC increased by more than two-fold, from 1.29% in 2009 to 2.75% in 2013 (p = 0.0001).

No perioperative mortality was observed. The follow-up visits took place within 4 to 6 weeks after the first surgical procedure was performed. All of the patients with a final histopathological diagnosis of TC were referred to the Center of Oncology Institute in Gliwice (Poland), and further treatment was conducted accordingly (resection of remnant thyroid tissue, lymphadenectomy if needed, and adjuvant radioiodine therapy).

## Discussion

The aim of every surgeon is to eliminate a disease while minimizing the risk of complications and minimizing or eliminating the necessity for reoperation. Secondary surgery of the thyroid has been established to carry a higher risk of complications than primary procedures, with worsening of prognosis for ITC patients. This issue is of particular concern for patients with MNG, which is the most common endocrine indication for surgical treatment [13–15,21–23,28–31].

Diagnostic procedures for TC performed before surgery for MNG are not completely reliable. Even preoperative FNAB often does not result in an accurate final diagnosis [25]. Therefore, TC is frequently an unexpected postoperative finding in MNG that is revealed only during histopathological examination. The incidence of TC is lower in patients with MNG than in those with a single nodule who have received thyroidectomy [29]. However, it is much more difficult to identify a malignant lesion in MNG [31]. Some authors have estimated that the incidence rates of malignancy in a single cold nodule and in a cold nodule in MNG are comparable [32]. The majority of patients with a small focus of papillary TC (i.e., smaller than 1.0 cm in diameter, with complete resection during first thyroid surgery) have a good prognosis. However, if histopathological examination reveals capsule invasion, multifocality or bilaterality, then the prognosis is worse [33–35]. Moreover, a TC focus may be detected in up to 10% of specimens of resected recurrent goiter if the first operation was performed for MNG [36]. The risk of reoperation due to recurrent goiter has been reported to be approximately 2% if all nodules are resected during the first operation; however, multinodularity of the thyroid gland is a risk factor for recurrence [37]. The estimated incidence of goiter recurrence after subtotal thyroidectomy varies from 13% to 20% among all patients, and regrowth of thyroid tissues occurs most often at 13 years after the initial surgery [13]. In 1965, Gould et al. concluded that there is no justification for total thyroidectomy for MNG when the risk of malignancy is low and the risk of postoperative complications is high [38]. However, primary total thyroidectomy for MNG is considered to be a safer procedure than reoperation [13,21,28,29,39]. This is why some authors, based of their own experience, recommend total or near total thyroidectomy for MNG to avoid a second operation in cases of ITC [26,40]. Further, some authors have reported that incidental papillary TC occurs so frequently in their population due to its high prevalence that it can be considered a normal finding [41]. In some surgical studies, incidental thyroid microcarcinoma has been detected in 2–24% of patients who have undergone surgery for MNG without any preoperative suspicion of malignancy. Further, multifocality is a very common characteristic of papillary thyroid microcarcinoma, occurring in 20–46% of patients [26,41].

The most common type of ITC, which is usually detected on histopathological examination of resected MNG specimens, is papillary carcinoma; however, some other types have also been observed. In our study, incidental papillary TC was diagnosed in 93.8% of all MNG patients with ITC. The other types, namely follicular and non-differentiated carcinoma, were found in 4% and 2% of the MNG patients, respectively. Yoshida et al. [42] presented 675 cases of anaplastic TC, 5% of which were incidental in nature. They noted that for this type of thyroid malignancy, only incidental anaplastic carcinoma was curable.

Small ITC is detected not only by physical examination, laboratory evaluation and several radiological imagining procedures but also commonly by UG-FNAB. Unfortunately, in some cases, ITC is clinically undetectable because of its small size and silent course. Such malignant thyroid foci are most often discovered on autopsy or in histological thyroid specimens removed due to presumed benign pathologies. Despite the improvement of diagnostic methods for TC (ultrasonography, elastography, and UG-FNAB with high-resolution transducers), which can detect thyroid nodules of between 1 and 2 mm in size, preoperative detection of small lesions is still insufficient [43]. One of the most unfavorable features of ITC in MNG is its small size, especially in cases of small ITC with a very aggressive course, as we described in one of our patients. Given the relatively high prevalence of ITC in MNG, it is apparent that more radical surgery conducted for this pathology would result in a lower risk of completion surgery, especially if total or near total thyroidectomy is performed. In our study, the prevalence rates of ITC detected in MNG patients after total or near total, subtotal and Dunhill resection were comparable. The most important clinical question is what proportions of ITC patients in these three groups require completion of primary surgery. In our study, we confirmed that more radical procedures for MNG are associated with a lower risk of reoperation due to ITC occurrence. This study has provided additional evidence in favor of conducting more radical surgical procedures for MNG. While each patient should be evaluated for surgery individually, in our opinion, total or near total thyroidectomy for MNG should be considered to avoid the necessity for reoperation due to the high prevalence of ITC. We acknowledge that the use of total strumectomy to treat MNG remains controversial; therefore, we propose a compromise, i.e., a less radical surgical approach for patients without bilateral nodularity or malignancy risk factors. Notwithstanding, the goal of our study was to emphasize the prevalence of ITC in patients without any established risk factors for malignancy.

According to the ATA Guidelines from 2015 [44], incidental thyroid microcarcinoma detected on histopathological examination of thyroid tissues removed for MNG or another benign disease does not require further treatment if total or near total thyroidectomy has been performed. Some of these patients must be treated more aggressively, e.g., those with ITC of an aggressive cell type, such as the tall cell or columnar cell variant of papillary TC [44,45]. A similar situation exists when a BRAF mutation or RET/PTC3 rearrangement is present [45,46,47]. In the 1970s, Simonowitz et al. recommended total thyroidectomy for patients who had previously received irradiation because of the high incidence rate of ITC [48]. Bradly et al. [16] have reported that papillary cancer occurs in 12% of resected thyroid tissues considered as benign, with the highest rate observed in Hashimoto's thyroiditis. Therefore, these authors have suggested that Hashimoto's thyroiditis increases the risk of incidental papillary TC. Further, incidental papillary TC has been detected in the contralateral lobes of 40% of patients undergoing surgery for follicular adenoma. This finding in MNG patients may favor total/near total thyroidectomy.

Another study suggesting a more radical approach was conducted by Untch et al. During their 10-year study period, they noticed that multifocal and unifocal contralateral cancers were present in 34% of patients who had undergone thyroidectomy completion for well-differentiated TC [49]. Karakoyun et al. [50] detected microscopic pathologic findings in remnant tissues after subtotal thyroidectomy in over half of their patients, and it was also contralateral to the dominant nodule. Moreover, ITC was present in 10% of the patients, with detection of papillary microcarcinoma in approximately 10% of residual thyroid tissues [50]. Gál et al. [51] performed 264 total thyroidectomies for MNG, focusing on patient outcomes and complications, to evaluate efficacy, safety, and histopathological findings. They found that the frequency of ITC was 3.5%. They did not observe any recurrence during an average of 6.2 years of follow-up. Based on multi-institutional experience with 1523 patients who had undergone surgery for Grave’s disease (N = 264), MNG (N = 1095) or toxic nodular goiter (N = 164), Smith et al. [52] have suggested that total thyroidectomy is the procedure of choice for the treatment of bilateral nodular disease. The rates of ITC in this study were higher than expected, reaching 18.3% in toxic MNG, 17.5% in MNG and 6.1% in Grave’s disease. The risk factors for ITC were male gender, the presence of thyroid nodules and a younger age. Thyroiditis and preoperative FNAB were not associated with cancer [52]. Costamagna et al. [53] have suggested that total thyroidectomy without residuum is a viable option for the treatment of benign thyroid lesions, including MNG, because of the increasing incidence of ITC, the eventuality of its multifocality and bilaterality and the potential for relapse [53]. Other authors are convinced that lobectomy is a sufficient surgical procedure associated with a very low risk of recurrence for incidental papillary microcarcinoma diagnosed after surgery if multifocality, extracapsular extension and histologically proven lymph node metastases are excluded [54].

An even less defined surgical approach has been established for ITC complicating Grave’s disease. Total thyroidectomy is considered an adequate and sufficient procedure for the treatment of TC in such patients. The prevalence of ITC in Grave’s disease is comparable to that in MNG, and it is higher than that in the general population. According to Ergin et al. [55] and Preece et al. [56], thyrotoxicosis does not provide protection against ITC.

Wilhelm has suggested other potential reasons for the increasing incidence of TC, including the increasing use of more precise diagnostic procedures, namely fluorine-18-fluorodeoxyglucose positron emission computed tomography (18F-FDG PET/CT), which provides a higher rate of thyroid incidentaloma detection [57]. These authors have suggested that focal high 18F-FDG uptake in the thyroid gland is associated with an increased risk of malignancy. In two independent papers, Brindle et al. [58] and Nayan et al. [59] have emphasized that up to 25% of these incidentalomas appear to be malignant. Chun et al. [60] and Yaylali et al. [61] have detected malignancy in thyroid incidentalomas by 18F-FDG PET/CT in 29% and 12.3% of cases, respectively, and they have confirmed that the positive predictive value of this procedure in malignancy of thyroid incidentalomas is 41.7%. They have concluded that patients with MNG and "negative" FNAB results but with high 18F-FDG uptake in the thyroid gland require a particularly careful diagnosis and radical treatment by total or near total strumectomy. Further, determination of the Tg level, along with the McGill Thyroid Nodule Score (MTNS), increase the sensitivity of detection of well-differentiated malignancy in patients with thyroid nodules (MNG) [62].

The risk of failure to detect cancer in South African MNG patients has been reported to be 6%, which is approximately three times higher than that determined in our study [63]. The most common of this type of incidental cancer in this previous study was the follicular variant of papillary carcinoma, whereas in our study, the classical variant was predominant.

The use of non-radical procedures to treat MNG results in a lower incidence of postsurgical complications than that observed with the use of radical procedures. However, some authors have suggested [64] that there is no significant difference in the incidence of postsurgical complications between radical and non-radical MNG surgery. The most frequently observed postsurgical complications are uni- and bilateral recurrent laryngeal nerve palsy and hypocalcemia; thus, during thyroidectomy, essential effort should be focused on safely preserving the blood supply of the parathyroid glands. Other authors have indicated that postoperative hypoparathyroidism and the resulting clinically and laboratory evident hypocalcemia are common complications of more radical surgery [65]. Further, Ozbas et al. have observed that the severity and exacerbation of postoperative hypocalcemia are directly and proportionately correlated with the extent of thyroidectomy [66]. However, these authors have reported that although early, transient, and clinically overt postoperative hypoparathyroidism depend on the extent of surgery, late, permanent hypocalcemia is not directly correlated with the extent of surgery. This group has also reported very similar rates of permanent hypoparathyroidism after radical and non-radical thyroidectomies. In contrast, radical surgery has been shown to protect patients from goiter recurrence, which occurs more frequently after non-radical procedures [8]. The risk of complications is higher in secondary surgery, which may be necessary if ITC is diagnosed postoperatively [44]. Notably, if radical surgery is performed, then secondary surgery is not required, even in cases of ITC. However, some authors have suggested that radical and non-radical procedures performed by specialists with experience in thyroid surgery have the same levels of safety. Although several studies have suggested that the incidence of postoperative complications is similar between radical and non-radical procedures [64,66–68], we believe that each type of surgery has some advantages and disadvantages. More radical procedures indeed may be associated with a higher risk of injury of the recurrent laryngeal nerve and parathyroid glands. However, because of the huge ampleness and great importance of these problems, i.e., post-thyroidectomy early and permanent complications, they will be analyzed and presented in a future, separate study.

## Conclusions

In our study, the incidence of TC diagnosed postoperatively in patients who underwent surgery for MNG without any previous suspicion of malignancy was lower than that previously described in the literature. The number of radical operations (total or near total) performed for MNG increased considerably over our analysis period, along with an increase in the prevalence of ITC in MNG. In the studied population, the prevalence of ITC in MNG was comparable between the patients who had undergone a primary radical procedure and those who had received a non-radical procedure. Due to the increasing incidence of ITC in MNG, total or near total thyroidectomy should be considered to avoid the necessity for reoperation. More radical procedures for MNG result in a lower risk of reoperation due to ITC.

## Supporting Information

S1 Dataset Data used in analyses. (XLS)
